# Immediate Effects of Instrument-Assisted Soft-Tissue Mobilization Versus Dry Needling on Trigger Point Pain and Tightness of Calf Muscles in Long-Distance Runners

**DOI:** 10.7759/cureus.57984

**Published:** 2024-04-10

**Authors:** Vaibhavi Pathade, Swapnil U Ramteke

**Affiliations:** 1 Sports Physiotherapy, Sancheti Institute for Orthopedics and Rehabilitation College of Physiotherapy, Pune, IND; 2 Sports Physiotherapy, Ravi Nair Physiotherapy College, Datta Meghe Institute of Higher Education and Research, Wardha, IND

**Keywords:** runners, calf tightness, pain, instrument assisted soft tissue mobilization, trigger point, dry needling

## Abstract

Introduction

Muscle tightness is frequently identified as a potential precursor to muscle injuries. Reclaiming flexibility and enhancing range of motion (ROM) is crucial for preventing injuries and achieving improvements in performance. The present study examines the immediate effects of instrument-assisted soft-tissue mobilization (IASTM) and dry needling (DN) in reducing trigger point pain and calf tightness in long-distance runners.

Methodology

A total of 40 long-distance runners were recruited in the study (30 males and 10 females). The procedure was performed under the author's surveillance at the sports complex. These recruited players were placed into two groups: the IASTM (n=20) and the DN (n=20) group. The outcome measures used were the pressure algometer for assessing pain pressure threshold and the lunge test. An iPhone Measure app (Measure app, Apple App Store 2023) is used to assess ankle dorsiflexion ROM. The evaluation took place both prior to and immediately following the intervention and 48 hours after the intervention.

Result

The analysis within each group revealed a significant alteration in pain pressure threshold for both the IASTM and DN groups (p≤0.05), along with a relative enhancement in ankle dorsiflexion ROM observed in the IASTM group (p≤0.05). Between-group analysis revealed a notable difference with an effect size difference of Cohen's d=1.06 (large difference) in pain pressure threshold, d=0.21 (small difference) in lunge test, and d=0.57 (medium difference) in ankle dorsiflexion ROM.

Conclusion

The present study concludes that both groups, IASTM and DN, showed significant effects in improving pain pressure threshold in long-distance runners. However, DN showed better results. IASTM showed significant results in enhancing the ankle dorsiflexion ROM immediately. This implies that it can be used in conjunction with stretching to decrease pain and enhance flexibility, hence improving performance and preventing injuries.

## Introduction

Running stands out as one of the most widely embraced sporting activities [1]. A systematic review by Kakouris et al. stated the occurrence and frequency of soft-tissue injuries related to running in long-distance runners were 40% and 44%, respectively, all of which were related to the lower limb, with the lower leg being the third most frequently injured site [2]. Adequate ankle joint range of motion (ROM) is essential for different functional activities like walking, running, and squatting [3]. Deficient ankle dorsiflexion may modify lower limb biomechanics during gait, potentially increasing the susceptibility to injuries. Restricted ankle dorsiflexion might impede the subtalar joint activity, hindering the ankle joint from attaining a stable close-packed position essential for walking and running. Enhancing ankle dorsiflexion, ROM is believed to be advantageous for runners as it may decrease the subtalar joint pronation, maintaining the joint closer to its optimal position. This, in turn, helps limit undesired movements that could contribute to any injury predisposition [4]. Muscular tightness is frequently viewed as a potential factor that predisposes individuals to muscle injuries. Limited ankle dorsiflexion ROM may contribute to restriction in gastrocnemius and soleus muscle flexibility. In runners, hamstring and calf muscles usually exhibit tightness, leading to hypertrophy of the gastrocnemius and hamstring muscle groups. This muscular stern is scientifically considered a potential contributing factor to injury [5]. Wang et al. found a 26% variation in ankle dorsiflexion ROM in runners and non-runners, with runners’ ankles being more restricted due to calf tightness [5]. Overuse of muscles may lead to the development of trigger points (TrPs) [6].

TrPs can emerge during vocational or athletic activities when muscle usage surpasses the muscle's capability and the regular recovery process is disrupted. Myofascial trigger points (MTrPs) are "hyperirritable spots, typically located within a tense band of skeletal muscle or in the muscle fascia. These points are painful upon compression and can elicit distinctive referred pain" [7]. The TrP is characterized by a tense band that heightens muscle tension and limits the ROM. Simons has presented the integrated hypothesis for MTrPs [8]. The instrument-assisted soft-tissue mobilization (IASTM), a widely used therapy for addressing myofascial restrictions, follows the principles introduced by James Cyriax [9]. It is a soft-tissue treatment method involving the use of an instrument to apply a mobilizing stimulus, aiming to have a beneficial impact on scar tissue and myofascial adhesion. There is a mechanical advantage to using instruments rather than a therapist's hands, enabling deeper penetration and potentially enhancing the precision of treatment application [10]. Simultaneously, this approach aims to reduce the tensity exerted on the therapist's hands during treatments. Trigger point dry needling (TrPDN) is a suggested invasive approach that has demonstrated effectiveness in alleviating pain associated with TrPs. Dry needling (DN) uses a fine, solid filiform needle [11] to penetrate the skin, subcutaneous tissues, and muscle [12]. There appears to be an evident gap in the literature concerning the lack of diversity in muscle studied, with most studies including the trapezius muscle. Presently, there is limited evidence concerning the acute effects of DN and IASTM on the function of the gastrocnemius and soleus muscles. Determining which method yields superior outcomes in a brief timeframe is crucial for clinical practice and during the competition phase, so this study aims to compare which technique would give faster and better outcomes on the calf muscle.

## Materials and methods

The present randomized controlled trial engaged 40 participants. Written informed consent was attained from the participants voluntarily before the initiation of the study. Ethical approval was obtained from the Institutional Ethical Committee of Sancheti Institute for Orthopedics and Rehabilitation College of Physiotherapy (approval number: IEC-SIOR/Agenda 070). The trial was registered at the Clinical Trials Registry-India (CTRI) on 03/02/2023 (trial registration number: CTRI/2023/02/049430). Eligible participants were randomly allotted into the IASTM group (n=20) and DN group (n=20) by computer-generated software. Outcome measures were gauged by sports physiotherapists who were blinded to the allocation. The therapist providing the intervention was blinded to the reading of the pre-intervention assessment and post-intervention assessment.

Inclusion and exclusion criteria

Long-distance runners were recruited from different sports complexes in Pune. Professional long-distance runners (marathon runners) who participate in competitions of 5000 m and above, age group of 18-25 years, runners having a minimum of one active TrPs in the calf muscle, and runners having decreased dorsiflexion (>10 degrees decreased) on iPhone Measure app [13] were enrolled in this study. Exclusion criteria were all the athletes who did not participate in long-distance running (5000 m and above), runners diagnosed with fibromyalgia, and runners who had any recent injuries or fractures of the lower limb [14]. For DN, the exclusion criteria were confined infection, high-risk pregnancy, allergy to metal, and patients on long-term anticoagulants and steroids [14,15].

Procedure

**Figure 1:**
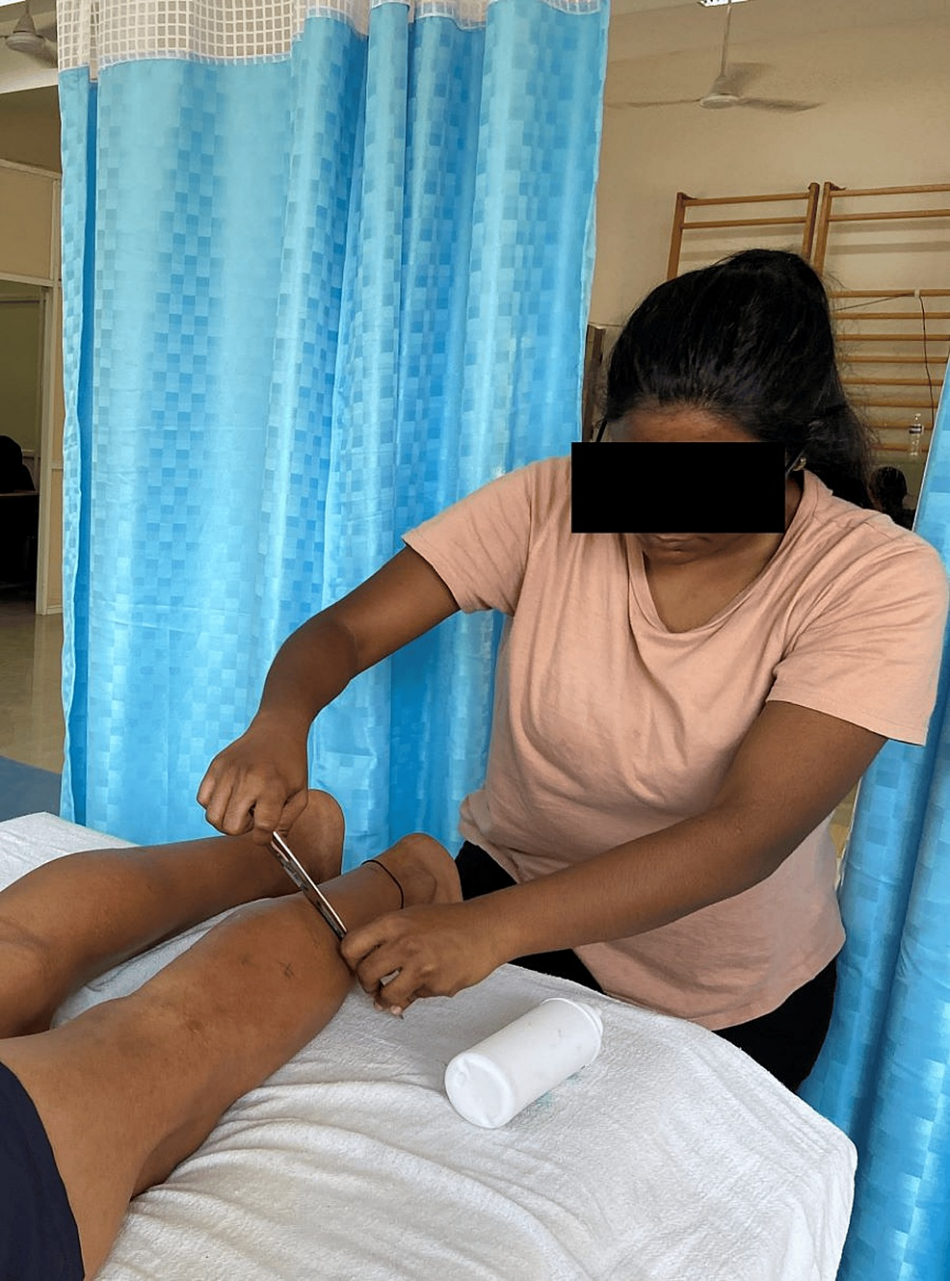
Therapist performing instrument-assisted soft-tissue mobilization for the calf muscle

The long-distance runners having TrPs and tightness in the calf were identified. Gastrocnemius and soleus muscles were assessed. Palpation was done to identify the taut band, which is located perpendicular across the length of the muscle fiber. A hyperirritable spot along the taut band was identified by sustained pressure for 10 seconds. The subject felt elicitation of symptoms, jump sign, radiating pain, etc. The TrP area was identified and marked on the calf muscle. A line was drawn from the mid-point of the popliteal fossa to the calcaneum and marked at 3, 6, 9, and 12 inches. The distance from the line to the TrP is at different inches according to the location of the TrP for reference in the post-48-hour assessment. The first intervention consisted of IASTM. The patient was lying in a prone position, and the calf area was exposed. The area was then prepared with lubricant. IASTM was performed across the entire length of the gastrocnemius-soleus complex with the IASTM tool. IASTM techniques using the tool were applied turn by turn over proximal and distal directions collateral to the muscle fibers on the structural tightness for two minutes. Figure 1 depicts the therapist performing IASTM for the calf muscle.

**Figure 2:**
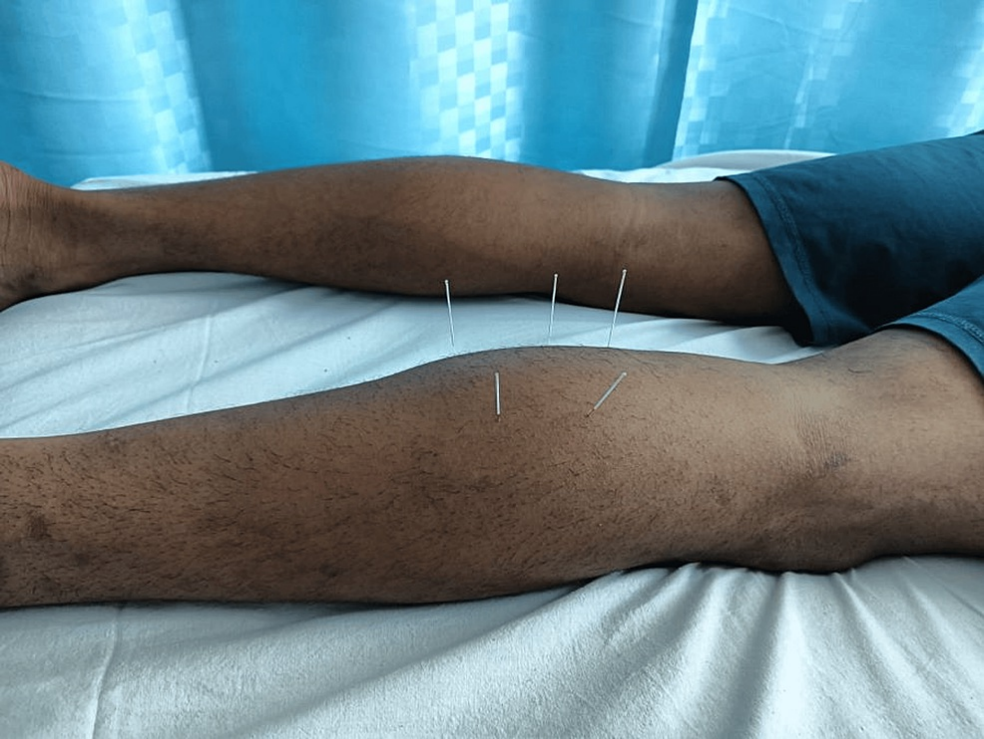
Dry needling for the calf muscle

The second protocol consisted of DN. The calf area was exposed in a prone position, and then the area was sterilized using spirit. The DN was carried out in the pre-assessed, palpated, marked TrP locations on the calf using sterile needles. Needles were inserted perpendicular to the skin across the affected calf. Subjects with DN as the interventional group received a solo session of TrPDN with disposable 0.25 × 40-mm stainless-steel needles. The needles were fixed in situ for 10 minutes [15]. Post-intervention stretching, both groups were instructed to stretch the calf muscle for 30 seconds and hold for three repetitions. Figure 2 shows the application of DN on the calf muscle.

The pain pressure algometer of the Orchid Scientific company was used on the marked spot. The algometer tip, which was 1 cm^2^ area, was firmly placed perpendicularly on the spot, and pressure was applied until the patient perceived the pressure as pain. The reading was recorded by taking three trials of the test, and the highest pain tolerance among the three was documented. Pain pressure threshold (PPT) was evaluated pre-intervention and post-48-hour intervention for both groups. The lunge test quantified the ankle dorsiflexion ROM. This test is done against the wall with standard tape measurements [16]. The lunge test was evaluated at three different timelines, i.e., prior to the intervention, just after the intervention, and after 48 hours of intervention, for both groups. For the assessment of ankle dorsiflexion, the application used was the iPhone Measure app. The iPhone was placed at the posterior lower limb, posteriorly in alignment with the TA tendon and calcaneum, with the iPhone showing 0 degrees in a neutrally placed ankle joint. Athletes were then conveyed to perform weight-bearing maximum ankle dorsiflexion in a lunge position. The iPhone was moved along with the leg, and the ROM was recorded [17]. Subjects were evaluated pre-intervention, immediately post-intervention, and 48 hours after intervention on the iPhone Measure app for both groups. Decreases in more than 10 degrees difference in total ankle dorsiflexion were taken as subjects.

Statistical analysis

IBM SPSS Statistics for Windows, V. 26.0 (IBM Corp., Armonk, NY) with an alpha value set at 0.05 was used. Baselines for the pre-intervention value of PPT, lunge test, and ankle dorsiflexion ROM were found to be matched, and the following tests were used. Within-group tests for non-parametric data, namely, the PPT values, were evaluated using the Wilcoxon signed-rank test. Parametric data, namely, the lunge test and ankle dorsiflexion ROM values, were evaluated using repeated measures analysis of variance to compare the pre-post and 48-hour data after the intervention in each group. For the between-group analysis, non-parametric data was evaluated using the Mann-Whitney U test for PPT. Parametric data were analyzed using an independent t-test for the lunge test and ankle dorsiflexion ROM.

## Results

This research involved 40 participants, evenly split into two groups of 20 each, with the mean age of Group A being 21.1 and that of Group B being 20.7. Initially, there were no notable variations between the groups (Table 1). Results of baseline matching which was performed with pre-values of Group A (IASTM) and Group B (DN) showed no significant difference in the outcome variables, i.e., PPT (p=0.231), lunge test (p=0.125), and ROM (p=0.210). However, upon examining each group separately, significant disparities emerged in PPT. Additionally, within the IASTM group, notable differences were observed in the lunge test and ankle dorsiflexion ROM. When comparing the two groups, a significant variance in PPT was identified, indicating a substantial effect. Moreover, improvements in ankle dorsiflexion ROM, assessed through the lunge test and iPhone Measure app, were notably greater in the IASTM group, showing a moderate effect size.

**Table 1:** Baseline demographic characteristics of the two groups PPT: pain pressure threshold; ROM: range of motion, IASTM: instrument-assisted soft-tissue mobilization; DN: dry needling

Characteristic	IASTM (n=20)	DN (n=20)	P-value
Age	21.1	20.7	0.580
Gender n (%)			
Male	16 (80)	14 (70)	-
Female	4 (20)	6 (30)	-
PPT	20.83±5.71	22.85±5.64	0.231
Lunge test	6.56±1.11	7.02±0.62	0.125
ROM (iPhone Measure app)	23.4±3.69	24.64±2.15	0.210

PPT comparison in both groups pre- and post-treatment

**Figure 3:**
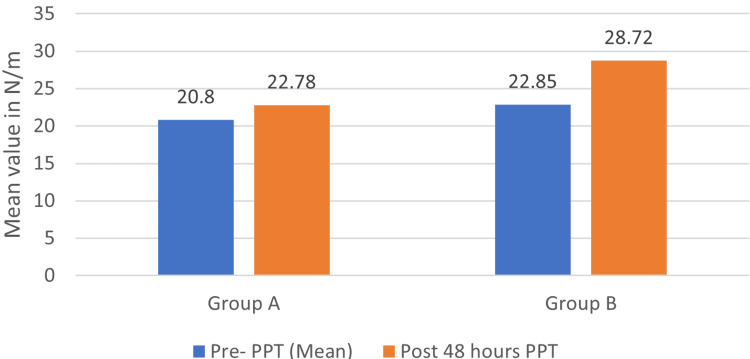
PPT comparison in both the groups pre- and post-treatment PPT: pain pressure threshold

In Group A (IASTM), the mean PPT on pre-intervention was 20.83±5.71, which was increased to a mean of 22.78±5.82 48 hours after intervention. The p-value by the Wilcoxon signed-rank test was found to be <0.001, which is statistically significant. In Group B (DN), the mean PPT on pre-intervention was 22.85±5.64, which was increased to a mean of 28.72±5.32 48 hours after intervention. The p-value by the Wilcoxon signed-rank test was found to be <0.001, which is statistically significant. Figure 3 shows a comparison of PPT in both the pre- and post-treatment groups.

Lunge test comparison of pre-intervention, post-intervention, and 48 hours post-intervention for both groups

**Figure 4:**
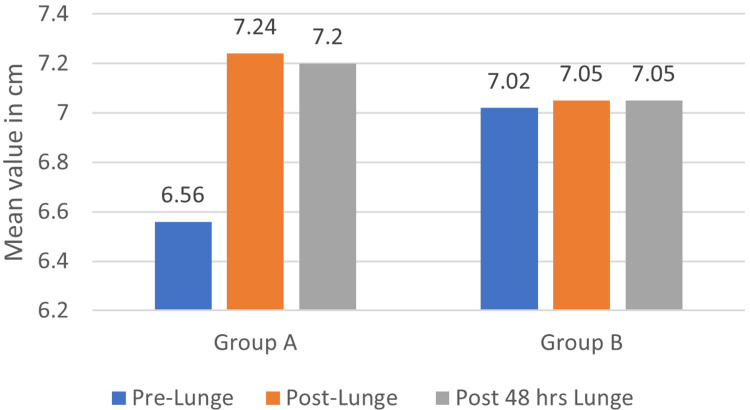
Comparison of lunge test pre- and post-intervention for both groups

In Group A, the mean lunge test value on pre-intervention was 6.56±1.11 which was increased to a mean of 7.24±1.07 immediately post-intervention and further reduced to a mean of 7.2±1.06. The p-value by the repeated measure test was found to be <0.001, which was statistically significant. The pairwise comparison of the lunge test between the pre-test and post-intervention showed a significant difference of p<001; similarly, the pre-test value and post-48-hour intervention also showed a significant difference of p<0.00. In Group B, the mean lunge test value on pre-intervention was 7.02±0.62, which was increased to a mean of 7.05±0.62 immediately post-intervention and was constant to a mean of 7.05±0.62. The p-value by the repeated measure test was found to be 0.248, which is statistically not significant. The pairwise comparison of the lunge test between the pre-test and post-intervention did not show a significant difference of p=0.248, and the pre-test value and post-48-hour intervention also did not show a significant difference of p=0.126. Figure 4 depicts a comparison of the lunge test pre- and post-intervention for both groups.

Comparison of ankle dorsiflexion ROM using the iPhone Measure app pre-intervention, post-intervention, and 48 hours post-intervention

**Figure 5:**
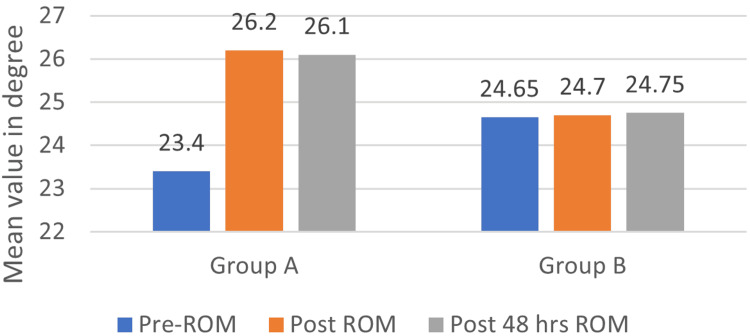
Comparison of ankle dorsiflexion ROM using iPhone Measure app pre- and post-intervention ROM: range of motion

In Group A (IASTM), the mean of ankle dorsiflexion ROM on pre-intervention was 23.4±3.69 which was increased to a mean of 26.2±3.04 immediately post-intervention and constant to a mean of 26.1±3.04. The p-value by the repeated measure test was found to be <0.001, which is statistically significant. The pairwise comparison of the ankle dorsiflexion ROM between the pre-test and immediate post-intervention showed a significant difference of p<001. The pre-test value and post-48-hour intervention showed a significant difference of p<001. In Group B, the mean of ankle dorsiflexion ROM on pre-intervention was 24.65±2.15 which was increased to a mean of 24.7±2.14 immediately post-intervention and constant to a mean of 24.75±2.14. The p-value by the repeated measure test was found to be 0.990, which is statistically not significant. In the pairwise comparison of the ankle dorsiflexion ROM between the pre-test and immediate post-intervention, there was no significant difference, p=0.990; the pre-test value and post-48-hour intervention also did not show any significant difference, p=0.488. Figure 5 depicts a comparison of ankle dorsiflexion ROM using the iPhone Measure app pre- and post-intervention.

Comparison of all the outcome measures for both groups

For PPT, Group B showed a large difference in effect size post-intervention, the lunge test showed a small effect size difference post-intervention, and there was no difference post-48-hour intervention. For ankle dorsiflexion ROM using the iPhone Measure app, a medium difference in effect size was seen post-intervention and 48 hours post-intervention. Table 2 depicts a comparison of all the outcome measures for both groups.

**Table 2:** Comparison of outcome measures for both groups PPT: pain pressure threshold; ROM: range of motion

Outcome measures	Group A (mean difference)	Group B (mean difference)	Effect size
PPT	1.95	5.87	1.065 (large difference)
Post-lunge	0.685	0.03	0.21 (small difference)
Post-48-hour lunge	0.64	0.04	0.16 (no difference)
Post-ROM (iPhone Measure app)	2.8	0.05	0.57 (medium difference)
Post-48-hour ROM (iPhone Measure app)	2.7	0.1	0.51 (medium difference)

## Discussion

The TrPDN is believed to reduce pain through several mechanisms. The fixation of the needle triggers muscle pain receptors that have become more sensitive, providing a mechanical stimulus. This action can diminish metabolic mediators and enhance local microcirculation. The swift reduction in pain and tenderness following TrPDN may be linked to the decline in chemical mediators [18]. Common causes of muscle tightness can be overloading and overtraining of the muscle without appropriate stretching and relaxation periods. In the current study, we used both the lunge test and ankle dorsiflexion ROM to measure calf tightness. A lunge test is performed with the knee flexed, hence more suggestive of soleus tightness and ankle dorsiflexion ROM measured via the iPhone Measure app with the knee extended, suggesting more gastrocnemius tightness. For the lunge test and ankle dorsiflexion ROM, we discovered a significant change post-treatment in the IASTM group, and the change was persistent after 48 hours of treatment. The DN group showed some effect post-treatment, but the effect was not statistically significant. The small effect shown may be due to the stretching of calf muscles.

It was reported by Lee et al. that both IASTM and the roller massage stick resulted in statistically significant enhancements in both active and passive ROM following a single treatment and these increases were still present 48 hours later, which concurs with our findings, that is, the calf length measured by lunge test and ankle dorsiflexion ROM was maintained after 48 hours post-IASTM [19]. Laudner et al. demonstrated an increase in glenohumeral (GH) horizontal adduction and internal rotation ROM in the IASTM group compared to the control group among baseball players [20]. Myofascial manipulation techniques may enhance the ROM by altering the fascia's density through the thixotropic property. This theory posits that applying energy like heat or mechanical pressure transforms the fascia from a denser "gel" to a more fluid state, increasing flexibility and ROM when stress is exerted on it [21-23]. This increase in ROM may help prevent injuries and/or also improve performance [24]. Both the lunge test and ankle dorsiflexion ROM did not demonstrate a significant change in the DN group, leading us to the conclusion that DN is not effective in decreasing both gastrocnemius and soleus muscle tightness in the present study. Contrastingly, a systematic review indicates an elevated cervical ROM following trapezius DN, revealing substantial between-group mean differences in effect sizes for nearly all assessed outcomes [18].

Other researchers have also noted an increase in ROM following DN, particularly in studies focused on the trapezius muscle, suggesting the potential efficacy of DN for this area [24,25]. The mechanism underlying DN involves applying localized stretch to shortened sarcomeres and contracted cytoskeletal structures within MTrPs. This process is believed to allow the sarcomere to return to its resting length by reducing the overlap between actin and myosin filaments [14]. Additionally, one study demonstrated increased knee extension after hamstring DN using the fast in-fast out technique [26]. Several other studies have shown positive ROM outcomes with the fast in-fast out technique [18,26,27], contrasting with our study where needles were left in place, indicating the potential superiority of the fast in-fast out technique. It's noted that during self-controlled lunging, some dancers in the DN group may have experienced soreness from the intervention, potentially restricting their passive motion [28]. Furthermore, Huguenin et al. found that both DN and placebo needling of the gluteal muscles did not result in any changes in straight leg raise or hip internal rotation [29].

The limitation of the present study was that it was of short duration, so the long-term effect on PPT and ankle dorsiflexion ROM was not monitored. Another shortcoming was the small sample size. For the DN technique, the needles were left in situ, rather than the fast in-out technique. Additionally, there was no control group in this study.

## Conclusions

The present study concludes that both groups, IASTM and DN, showed significant effects in improving PPT in long-distance runners. However, DN showed better results. IASTM showed significant results in improving the ankle dorsiflexion ROM immediately. Therefore, it can be used in conjunction with stretching to decrease pain and enhance flexibility, hence improving performance and preventing injuries.
